# Chitosan-Based Hydrogels in Vascular Tissue Engineering Applications

**DOI:** 10.3390/ma19132715

**Published:** 2026-06-24

**Authors:** Lauren Taylor, Shih-Feng Chou

**Affiliations:** 1 Department of Mechanical Engineering, College of Engineering, The University of Texas at Tyler, Tyler, TX 75799, USA; ltaylor36@patriots.uttyler.edu; 2 Advanced Materials and Manufacturing Institute, College of Engineering, The University of Texas at Tyler, Tyler, TX 75799, USA

**Keywords:** chitosan, hydrogels, vascular repair, polymer cross-linking, stents

## Abstract

The development of biocompatible materials has gained traction due to the increasing clinical demands for customizable and functional medical devices. Chitosan, a deacetylated derivative of chitin, is a naturally occurring biopolymer with strong antimicrobial properties, immunocompatibility, and structural adaptability, making it a promising candidate for biomedical applications. Through mechanisms such as crosslinking, ionic bonding, gas formation, and UV radiation, the mechanical properties and stimulus responses of chitosan-based hydrogels can be tailored for drug delivery at specific sites or under specific pH, light, or electrical conditions. Beyond drug delivery, chitosan hydrogels have shown considerable potential for vascular tissue repair. The porous structure of chitosan allows patient specific vascular scaffolding to be created that promotes the recovery rate veins and stenting procedures. Thermally sensitive hydrogels can deliver drugs to target regions to further assist in vascular healing. Furthermore, recent developments with composite polymers and coatings engineered to self-assemble within veins provide scaffolds for vascular tissue growth. This manuscript reviews chitosan hydrogel fabrication methods and their corresponding materials properties, with particular emphasis on drug delivery to vascular tissues. Furthermore, relevant findings from clinical trials are summarized to support the potential of chitosan hydrogels for future clinical use. Challenges of chitosan hydrogels, such as insufficient mechanical strength, high degradation rates, and complex manufacturing, remain as areas for research break-through.

## 1. Introduction

Biomaterials have found extensive use in modern biomedical applications, including drug delivery, vascular tissue engineering, and medical devices. These materials include a broad range of natural and synthetic materials, such as metals, ceramics, and polymers, which each offer distinct advantages depending on the application. Among them, polymers have attracted particular interest due to their ability to have variable characteristics and biocompatibility. Both natural and synthetic polymers are studied, but several of these polymers have limitations. For example, the natural polymer collagen can lack the mechanical strength needed for vascular tissue, while the synthetic polycaprolactone faces challenges in fabrication [1]. Another synthetic polymer, polyethylene glycol, demonstrates favorable hydrophilicity and biocompatibility but can result in immune responses during treatment [2]. Other polymers include alginate, which lacks cell adhesion peptides, and gelatin, which can be challenging to modify due to changing viscosity with temperature [3]. In this context, chitosan has emerged as a promising biocompatible polymer due to its intrinsic biocompatibility, antimicrobial activity, and ease of chemical modification. Furthermore, chitosan can be used both as a standalone material and in composites to strengthen performance in vascular applications.

**Figure 1:**
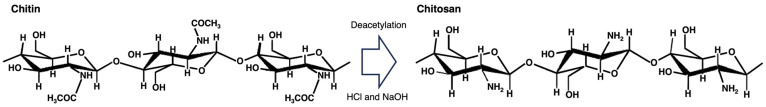
Chemical structures of chitin and chitosan showing the deacetylation process.

Chitosan, a derivative of chitin, is a naturally occurring biopolymer found abundantly in nature from living organisms and can also be synthesized from biological sources. Chitin, the precursor of chitosan, is found in the exoskeleton of crustacea, insects, algae, and some types of fungi [4]. Chitin’s molecular structure consists of a copolymer of d-glucosamine and *N*-acetyl-d-glucosamine. Deacetylation of chitin removes acetyl groups while producing d-glucosamines and acetates that represent chitosan [5], as shown in Figure 1. The number of acetyl and amine groups on the polymer chain determines the difference between the two biopolymers. For example, a molecular structure with 100% of amine groups is chitin, whereas one without the amide groups is called chitosan, with 50% amide groups being the boundary between the two.

Chitosan exhibits a highly ordered fibrillar structure with the ability to bind with negatively charged fat, ions, proteins, and other body structures [6]. Hemmami et al. reviewed the surface morphologies of the chitosan from various chitins, which is often referred to as chitosan’s microstructure, using a scanning electron microscope (SEM) [7]. For example, chitins obtained from house crickets, grasshoppers, and orthopteran species exhibited both nanofiber and nanopore structures [8]. On the other hand, chitin and chitosan from aquatic insects, water-scavenger beetles, desert locusts, and Colorado potato beetles were nanofibrous [9]. Moreover, chitin obtained from crabs and squillas demonstrated sponge-like surface morphologies [10]. The ability to visualize chitosan’s microstructure provides direct information on how it will behave. For example, chitosan with a higher molecular weight and a lower deacetylation degree tends to result in antioxidant properties and a higher water binding capacity, which influences the quantity of medicine that can be absorbed. Chitosan has been observed to extend the shelf life of fruit and vegetables with less pigmentation than control samples [11]. It is also effective in forming films used to create protective coatings against heavy metal substrates and has shown to have the ability to chelate metals such as copper, aluminum, and silver within the human body with future potential for helping extract toxins from the body [12].

Chitosan can be combined with other biopolymers and/or synthetic polymers to form blends with improved properties. When blended with alginate, a natural polysaccharide extracted from algae and bacteria, it displays strong rheological characteristics [13]. Complex interactions between the polysaccharides result in the formation of a more stable network structure with improved response to external forces. These networks are formed without direct chemical crosslinking and are instead result from the wrapping of structures which result in increased mechanical stability and viscoelasticity. Similar results are observed when chitosan is blended with hyaluronic acid, which increases the Young modulus [14]. Further property modifications can be achieved by blending the chitosan with collagen to improve the swelling ratio and increase the degradation time of the hydrogel. Collagen I, a subtype of collagen found in the human body, can also be used to improve the elastic properties and decrease the maximum stresses experienced by the hydrogel [15]. Studies have shown that the stability of chitosan and collagen blend hydrogels can be extended to over 14 days with a 75/25 combination even when exposed to human blood enzymes [16].

Some challenges facing the development of chitosan hydrogels are caused by the same properties that make them so appealing—namely being highly vulnerable to environmental changes. Hardness and strength were reduced when the raw product was exposed to temperatures below 40 °C [17]. In addition, storage of the hydrogels at ambient temperatures resulted in the complete degradation of the hydrogel as early as seven days [18]. High humidity contributed to a decrease in physio-chemical properties due to structural relaxation from water penetration and swelling [19]. For example, chitosan is incompatible with most thermoplastic bio-based polymers such as polylactic acid and polyhydroxyalkanoates due to the hydrophilic nature of chitosan which results in an inhomogeneous mixture [20]. In addition, while chitosan can be mixed with other biopolymers, there are many incompatible combinations that can decrease the mechanical properties. For example, blending chitosan with polyvinyl pyrrolidone were observed to cause no porosity in the structure. Furthermore, blending with polycaprolactone demonstrated decreased mechanical stability at low weight percentages of chitosan due to phase separation [21]. To overcome this limitation, further mixtures were created with various inorganic/organic nanoparticles which used their inherent zeta potential to optimize emulsions and films. This approach leads to a greater dispersion and stability of the polymer above its base properties [22]. Proteins can also be added into the structure, such as fibroblast growth factor 2 (FGF2), which is a critical protein for cell growth, differentiation, and vascular repair [23].

In this review, we aim to provide a comprehensive overview of chitosan hydrogels and their applications in vascular tissue engineering and drug delivery. The review examines physical, chemical, and biological properties of chitosan that make it suitable for vascular applications. Furthermore, fabrication methods and modification approaches to alter hydrogel characteristics for individual applications are covered. Findings are summarized from relevant preclinical and clinical studies for drug delivery and vascular applications. Finally, current challenges in mechanical strength, degradation, and fabrication are discussed to identify future research directions and support clinical adoption of chitosan hydrogels for vascular applications.

## 2. Deacetylation, Crosslinking, and Formation of Chitosan Hydrogels

### 2.1. Deacetylation of Chitosan

Chitosan is typically produced by the deacetylation of chitin, a chemical process in which the acetyl (-COCH_3_) groups on chitin are removed resulting in the formation of amine groups (-NH_2_). Depending on the degree of deacetylation, this process yields a block copolymer of *N*-acetyl-glucosamines and glucosamine with alternating length that determines the properties and type of chitosan. It is commonly accepted that chitosan exhibits over 50% of glucosamine units. Multiple methods have been developed to facilitate the deacetylation process of chitosan, including alkali treatment, enzymatic deacetylation, and steam explosion [24].

Alkali treatment using an aqueous sodium hydroxide (NaOH) solution on chitin is the most commonly used method to obtain chitosan. During the reaction, insoluble chitin is added to the NaOH solution, where the NaOH acts as a nucleophile, a chemical species that forms bonds by donating an electron pair and attacks the acetyl groups in the chitin to begin converting them to chitosan. However, the water content must be carefully managed since insufficient hydration of the NaOH solution yields a slow or negligible reaction. A secondary slowdown in reaction rate is associated with an increase in hydration of the chitin or the formation of a stable amide anion of chitosan which prevents further attack of the chitin macromolecule. Concentrations of NaOH solutions ranging between 20% and 60% have been reported to increase the degree of deacetylation of chitosan, while diluted NaOH solutions made the deacetylation process inefficient or ineffective [25]. Temperature is another critical factor that accelerates the reaction and improves the degree of deacetylation. Studies showed that the degree of deacetylation increased from 28% using 7.5 mol/L NaOH to 70% with a NaOH concentration of 12.5 mol/L while the reaction temperatures increased from 40 °C to 120 °C [26]. Results suggested that the deacetylation process was faster at a higher temperature due to the reduction in the required activation energy and achieved the same results as the reaction containing 12.5 mol/L or more NaOH.

Another method of deacetylation is through the use of enzymes on chitin. This can be performed with 0.5 µM of chitin deacetylase, an enzyme that catalyzes the removal of acetyl groups from chitin while using a Tris-HCl buffer to maintain a pH of around 8 throughout the reaction. Together with a cobalt chloride solution, the mixture was monitored after five days to ensure all available chitin had reacted fully [27]. Only α- and β- chitin (sourced from exoskeletons and some squids respectively) can be processed with this enzyme with an increase in degree of deacetylation of 10%, while γ-chitin, found in some beetles and squids, is unable to complete the deacetylation process successfully. The enzymatic action is unable to overcome the required energy to unpack crystalline chitins, due to the strong inter- and intra-molecular hydrogen bonds, to access all the *N*-acetyl groups.

Another method involves a pre-treatment of 18% formic acid, which is added to the chitin (10% degree of deacetylation) to cause faster precipitation to 90% DD chitosan. The chitin was demineralized using 18% formic acid with 1% of calcium citrate before being added to a chitin deacetylase solution, an enzyme used for the deacetylation of chitin, at 50 °C for 24 h. This method requires the selection of appropriate enzyme sources (e.g., fungi and bacteria species) that react efficiently with different sources of chitin. The enzymes can catalyze reactions on more than one molecule of chitin at a time and affect all available polymer ends. This multichain mechanism allows the enzyme to bind and remove acetyl groups in a sequential, block-wise manner, resulting in a high-precision approach with improved yield [28].

To improve deacetylation of chitin using enzymatic methods, an alternative approach is the use of sonification and steam explosion methods. The process involves subjecting the chitin (0.1–2 g/mL) to steam generated from deionized water at 180 °C and 1 MPa, followed by sudden release of pressure to the atmosphere after a residence time of 1 to 8 min. By using high-pressure steam, the rigid structure of chitin is disrupted, where nanopores are formed with a reduction in density and crystallinity of chitin to allow enzymes or other chemicals to penetrate the molecule structure further. Sonification was used to acquire the structural changes that determine the effectiveness of the degree of deacetylation. In a study, the degree of crystallinity was lowered by 11.28% and could be used on both chitin and chitosan to increase the degree of deacetylation to 96% [29].

### 2.2. Chemical Crosslinking

The development of medical devices for treatments in human bodies using biological materials requires a high degree of precision due to incompatibility issues and the unwanted adverse reactions. Chitosan is a natural cationic copolymer, a polymer that is formed by combining two or more monomers where at least one of which carries a positive charge. Due to this natural mixture, it is readily soluble in acidic aqueous solutions and is biodegradable by human enzymes. Chitosan’s hydrophilic nature facilitates the formation of hydrogels that absorb significant volumes of water. Crosslinking occurs within the polymer, trapping water inside, and its density is inversely proportional to the quantity of water in the hydrogel. One crosslinking method is to dissolve chitosan in an acidic medium to generate hydrogen bonds and therefore crystalize the structure [30]. A straightforward method for crosslinking chitosan is the use of dedicated crosslinker chemicals, including genipin, epichlorohydrin, glutaraldehyde, and sodium tripolyphosphate [31]. Once chemically bonded, the operation is irreversible and results in a stable compound which yields properties of high mechanical stability and low reactivity with environmental changes [32].

Genipin-crosslinked chitosans were prepared using a 0.5% (*w*/*v*) genipin solution with a reaction time of over 15 h at room temperature [33]. After the reaction, an acetate buffer (0.1 M), NaCl (1M), and ethylene glycol (30% *w*/*v*) were added to eliminate ionic and hydrophobic interactions between the chitosan particles and enzymes. The buffer was used to prevent reversible hydrophobic reactions that reduce the quality of the chitosan and improve access to the active site of the reaction. Thermal stability was observed up to 250 °C, but significant weight loss began at 100 °C with between 25.8% and 30.8% of the mass lost. The change was attributed to the crosslinked chitosan having lower thermal stability and weakening of the structure causing complex dehydration of the saccharide rings and depolymerization.

Epichlorohydrin crosslinking starts with immersing chitosan in a solution of 0.1–1.0 M epichlorohydrin and 0.067 M NaOH. It is suspended for two hours with constant stirring and heat at 30 °C. The final product is then filtered, washed with distilled water, dried, and stored in a desiccator to remove any unreacted chemicals prior to storage. This method produced a final product that is white in color and translucent when dry and produced a gel with a swelling ratio between 0.6% and 2.8% depending on epichlorohydrin concentration. This serves as a metric to compare drug loading capacities of the hydrogels, where hydrogel swelling after contact with the solution stabilizes after 10–15 min of submersion [34].

Chitosan can be crosslinked using glutaraldehyde, a widely used reagent that has been previously used for pharmacological and enzyme immobilization studies. In a study, 1.5 g batches of chitosan were dissolved in 20 mL of a NaOH solution and stirred for 24 h before adding 1.0–2.0 mL of a glutaraldehyde solution at room temperature [35]. The solutions were then cast and dried for 12 h to remove any residual solvents. When performing Fourier-transform infrared spectroscopy, the N-H wavelength vibration moved from 1652 cm^−1^ to 1635 cm^−1^ which is attributed to the effective crosslinking and electrostatic interactions between chitosan and glutaraldehyde. The swellability of the crosslinked chitosan decreased from 120% to 45–60% depending on the amount of glutaraldehyde added. This is a result of the crosslinking with glutaraldehyde making the gelled matrix more compact and reducing the water that can penetrate the structure.

In a study by Bhumkar and Pokharkar, chitosan was successfully crosslinked through the use of a 2.0% *w*/*v* solution of chitosan/NaOH mixed with a 0.1% *w*/*v* solution of sodium tripolyphosphate [36]. When creating this crosslinked chitosan, it was discovered that the final swelling properties of the chitosan were highly dependent on the pH of the sodium tripolyphosphate solution used to create it. This swellability ranged from 668.85% at a pH of 3 to a much lower 157.65% at a pH of 9. Swelling varied with changes in pH, which modified the crystalline nature of the chitosan. This caused an increase in polar groups, which also contributed to an increase in water-holding capacity that was a side effect of the pH changes.

Chitosan crosslinking was also performed by using a three-component method, which is a blend using glutaraldehyde, NaOH, and chitosan. The crosslinking process is performed in stages, with the base chitosan submerged in a 25% (*w*/*v*) solution of glutaraldehyde. It is then immersed in a 2 M of NaOH solution, washed, and dried at 37 °C. The chitosan is then immersed into a 1.3% (*w*/*v*) solution of sodium tripolyphosphate for one hour at 4 °C, then dried at 60 °C under vacuum to produce the final product. This method increases the pH stability in comparison to the non-crosslinked chitosan. Base chitosan swelled and disintegrated at a pH of 1.2, while the newly crosslinked product experienced stability in all tested buffer solutions (1.0–9.0 pH) [37].

Overall chemical crosslinking remains an effective approach for improving the mechanical properties of chitosan hydrogels. However, the potential toxicity of crosslinking chemicals and need for purification of the final products are limitations to the process. Glutaraldehyde is particularly promising due to its increased pH stability.

### 2.3. Formation of Chitosan Hydrogels

#### 2.3.1. Physical Crosslinking

In a study by Xu et al. [38], chitosan was sequentially filtered through a 0.2 μm cellulose acetate membrane, centrifuged and rinsed, and this was repeated until the neutral pH of 7.0 was achieved, resulting in chitosan with a viscometric molecular weight of 187 kDa. Acetic acid was then added and frozen within a mold at −20 °C to initiate gelation of the hydrogel. During sequential gelling, the properties of the chitosan hydrogels can be controlled by modifying the gelation time and composition. One limitation of this method is when the sample setting time is less than 24 h, which results in mechanical instability as demonstrated by rupture under tensile stress. Furthermore, additional gelling processes with the same sample yielded no significant change in properties. This method relies heavily on ionic crosslinking with tripolyphosphate groups to gel the chitosan. However, changes in rate, concentration, and/or temperature could cause a precipitate to form. Some of these major results from the study are summarized in Table 1.

A second sequential gelling method uses di-functionalized polyethylene glycol which is prepared as a benzaldehyde termination reaction of the base polyethylene glycol. This process begins when 100 mL of tetrahydrofuran is added to a flask and dissolves the glycol. Then, 4-carboxybenzaldhyde, 4-dimethylaminopyridine, and *N*,*N*′-dicyclohexylcarbodiimide are added sequentially. After drying for 12 h at room temperature, white solids are produced and dissolved in a cold diethyl ether bath, before filtering out any insoluble white solids. Separately, 0.11–0.44 g of the di-functionalized polyethylene glycol (DF PEG) is added to 5.0 mL of deionized water and 0.495 g of glycol chitosan. After adding the two chemicals together, a hydrogel was formed within a couple of minutes. The shear strength can be tuned by adjusting the quantity of DF PEG with 4.4 mg leading to a softer 900 Pa strength and 17.6 g resulting in a stiffer hydrogel at 4700 Pa strength [39].

Solutions of a chitosan and collagen mixture were prepared using 1.5 wt% concentration in acetic acid followed by pH adjustment from 3.2 to 7.0 using NaOH, along with temperature adjustment [40]. Viscosity changed significantly from 1000 Pa*s to 0.1 Pa*s as the shear strain rate increased from 0.1 s^−1^ to 100 s^−1^. Other studies used sodium chloride (NaCl) and phosphate-buffered saline (PBS, pH = 7.4) as solvents to replace strong bases in the formation of chitosan hydrogels [41]. These results demonstrate the importance of solvent/buffer solution in the gelation control of chitosan hydrogels, which also affected the biocompatibility of the chitosan hydrogels and the bioactivity of encapsulated agents.

When developing further experiments to determine the ideal chemical to combine with chitosan, biocompatibility is the most important factor. While cost effectiveness is a concern, the focus remains on achieving optimal biocompatibility. The mass swelling ratio observed was up to 60 times the original hydrogel mass which can be repeated for each chemical combination. The previously prepared collagen buffer solution was found ineffective in supporting cell adhesion due to its solubility in other buffer solutions, particularly in acidic solutions such as HCl and acetic acid, which are used in cell tissue experiments. Therefore, alginate was incorporated into the framework of chitosan and crosslinked by mixing 100 mL 0.5 M acetic acid with 2 g of chitosan powder and stirred for 25 min [42]. At this point, 50 mL of alginate stock, prepared with 2 g of sodium alginate in 100 mL of distilled water, was added drop by drop while stirring the chitosan solution, along with the same amounts of catalyst T-12 and DCC. This resulted in a hydrogel with hybrid polymeric fibers that successfully supported cell attachment.

Another type of chitosan hydrogel is produced by enzymatic crosslinking which uses enzymes as reagents to cleave or form covalent bonds. This method offers advantages due to lower reaction times and better control over hydrogel formation by controlling enzyme concentration. Tyrosinase, a copper-containing oxidase enzyme, was shown to be more than double the adhesive strength of crosslinked hydrogels [43]. In a study, 1 g of a chitosan and glycolic acid solution was dissolved in water after which a 15 mL solution of dimethyl sulfoxide containing tyrosinase was added [44]. Ultra-filtration, freeze drying, and evaporation were used to remove any remaining solvent before being diluted with water. Finally, any remaining residue was diluted with water and neutralized with a NaOH solution to a final pH of 7.

Physical crosslinking provides a viable approach in the production of chitosan hydrogels through the minimization of chemical agents or using natural materials. Physical crosslinking encounters limitations in mechanical properties, especially when using sequential gelling. However, these methods show promise in applications where variable mechanical properties or biocompatibility is desired.

#### 2.3.2. Stimulus-Responsive Hydrogels

Chitosan hydrogels can be produced via photopolymerization in combination with chemical crosslinking to improve the function of the polymer. This process can lower the curing time to less than a minute at room temperature rather than 24 to 72 h [45]. A grafting reaction was performed using glycidyl methacrylate monomer underneath a nitrogen atmosphere and the resulting chitosan hydrogel was precipitated out of solution into a white solid as its solubility decreased from the hydrophilic methacrylate groups. Previous thermal stability issues observed with chemically crosslinked chitosan, such as glutaraldehyde crosslinks at 205–300 °C and genipin crosslinks at 280–320 °C, were resolved after UV-induced polymerization, achieving thermal stability up to 430 °C due to improved crosslinking and attractive molecular interactions. After exposing the structure to UV light, swelling ratios up to 600% higher were observed. The resulting compound was observed to be less toxic or harmful than the chemically crosslinking process since it includes the same base materials and was confirmed with a cytotoxicity test.

The use of UV light for manufacturing a polymer adds the possibility of higher production levels with controlled systems using pumps [46]. A water phase of 1% (*w*/*v*) CS-nbn-COOH solution was added to the crosslinker, a short bifunctional thiolated-diethylene glycol, and pumped through a UV-permeable tubing wrapped around a domestic UV lamp. The flow rate was adjusted to achieve a residence time between 4 and 15 min and the solution was collected at the end in a beaker and recovered by centrifugation at 7000 rpm for 3 h at 6 °C. When using CS-nbn-COOH (norbornene-derived chitosan polymer) with concentrations between one and two percent, instant gelation was achieved with exposure to the UV light. A mechanical stirring system enhanced reproducibility, enabling the construction of a predictive regression model with a coefficient of 0.94.

Hydrogel pH sensitivity was enhanced through the addition of 2-hydroxyethyl polymethacrylate [47]. For the preparation of the photopolymerized chitosan hydrogel, N-carboxyethyl chitosan aqueous solution was added to a solution of 2-hydroxyethyl polymethacrylate containing tetra(ethylene glycol) dimethylacrylate, and the photo-initiator Darocur 2959. Finally, the hydrogel was formed after transferring the solution into a round mold and irradiated with a 50W miniature UV arc lamp for 10–30 min, followed by drying under vacuum at 60 °C for 48 h. The pH sensitivity was highly dependent on the -COOH groups and their interaction with the surrounding pH. At pH levels above 9.0, the -COO carboxylate groups increased in concentration which led to higher osmotic pressure and swelling (swelling ratio increased from 1.0 to 1.9). Furthermore, at lower pHs between 2.0 and 4.0, the -NH_3_ groups drove the swelling rate up through the same osmotic pressure increase (swelling ratio increased from 1.0 to 1.6). The hydrogel swells under acidic conditions due to increased electrostatic repulsion between cationic chains and shrinks under basic conditions. The hydrogel is exposed to control pH solutions until a consistent behavior is achieved, and the deflection from swelling is used to detect as small as 1 × 10^−5^ changes in pH within the human body. With the high energy produced by the UV light, the amount of crosslinking agents is minimized and improves biocompatibility by preventing the use of toxic additives which may not have fully reacted and remain within the hydrogel [48].

Light-sensitive hydrogels are those that respond upon exposure to light stimuli. In this method, photochromatic particles are embedded into the hydrogel and some present swelling behavior due to solar heating [49]. To produce a light-sensitive hydrogel, groups such as azobenzene, diarylethene, and spiropyrans are incorporated covalently or non-covalently. Some such as poly-azobenzene, when introduced, not only produce enhanced room-temperature phosphorescence but also have self-healing properties. Two pieces of hydrogel were able to heal within one minute by isolating oxygen and stabilizing the structure. Following UV irradiation, the hydrogel exhibited shape memory behavior for wavelengths under 365 nanometers that would manually trigger the self-healing. When irradiation reached under 254 nm, the crosslinks are instead cleaved, resulting in a less rigid hydrogel due to the partially broken chemical bonds [50].

An additional trigger that can be built into hydrogels is pH sensitivity. Hydrogels can contain partially ionizable acidic units, which will become negatively charged with increasing pH, thereby causing their counter-ions to induce an increase in osmotic pressure. Tests must be performed to ensure that the swelling of the hydrogel does not cause the structure to swell and rupture in an autolysis process. Hydrogen bonds formed lead to rigidity at room temperature but can then be destroyed if their pH reaches outside of their ideal range of 3 to 10. Metal ligands, molecules bonded to a central metal atom, use changes in electron density to maximize the breaking stress of the hydrogel at specific environments [51].

Stimulus-responsive hydrogels have a unique advantage because they can dynamically respond to environmental conditions such as pH, temperature, and light. These hydrogels show strong potential in applications such as drug release and targeted treatments where the hydrogel must be released under specific conditions. Furthermore, mechanical properties can be adjusted through variations in the crosslinking process, and biocompatibility can be increased with methods such as UV crosslinking.

#### 2.3.3. Cryogels and Freeze–Thaw Systems

Performing freezing–thawing with four to six cycles down to −80 °C is an effective way of creating chitosan hydrogels [52]. Due to freezing, water molecules within the polymer structure transform into ice crystals, where thawing of these ice crystals leaves behind voids in the structure. This effect results in a porous microstructure devoid of entrapped gas bubbles. A decreased swelling capacity was observed with higher weight retention and up to 30 h of the anti-inflammatory drug diflunisal delivery occurred. No crosslinking chemical is required in this method and thus any adverse effects caused by them are avoided. However, a key limitation of freeze–thaw hydrogels is the requirement to maintain subzero storage temperatures prior to drug loading; premature thawing may compromise structural integrity and lead to mechanical failure. This limitation can potentially cause challenges during large-scale implementation and transport.

**Figure 2:**
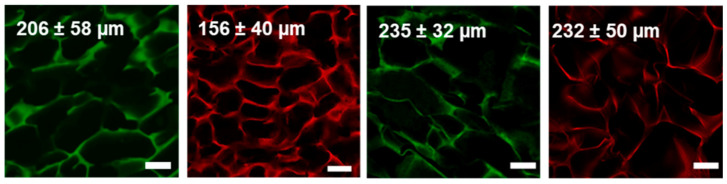
Confocal laser scanning microscopic images of chitosan (green) and carboxymethyl chitosan (red)-based cryogels crosslinked with 1,4-butanediol diglycidyl ether (**left**) and poly(ethylene glycol) diglycidyl ether (**right**) showing the average pore sizes. Scale bar = 100 µm. Figures were obtained from an open access article [54], distributed under the Creative Commons Attribution License.

Cryogels are another method of forming chitosan hydrogels that produces inherently interconnected structures at low temperatures without the need for intensive chemical washing [53]. As the solvent crystallizes, the chitosan is concentrated into the liquid microphases, which accelerate the rate of gel formation. After 2 to 3 h of crystallization, the cryogels are returned to room temperature. The solvent melts and hydrates the interconnected network, producing the final hydrogel structure. Due to the surface tension of the solvent, the pores in the cryogels became rounded rather than sharp edges. In one study, 3% chitosan solutions were prepared in HCl with pH adjusted to 5 using 0.1 M NaOH solution. The chitosan solutions were frozen at −10 °C for 12 days followed by thawing and washing with distilled water to create chitosan cryogels, where the porous structures are shown in Figure 2 [54]. Others prepared 0.55%, 1%, and 1.5% chitosan cryogels by dissolving chitosan in 2% acetic acid solution followed by crosslinking with a 0.5% glutaraldehyde solution [55]. The mixed solutions were frozen at −22 °C for 24 h, and the resulting cryogels were thawed at room temperature followed by washing and lyophilizing at −55 °C for 24 h. Both cryogels and freeze–thawing produce hydrogels with a porous microstructure which have potential in improving drug delivery while reducing the need for large quantities of chemical crosslinkers.

#### 2.3.4. Composite Hydrogels

Taking a more in-depth look at composite structures, one method is the use of multivalent ions to change the structure. These are ions that can exist in more than one oxidation state with the ability to carry different positive or negative charges. Pentasodium tripolyphosphate was used to increase the swelling ratio of the hydrogel from 100% up to 1125% after half an hour in a buffer solution with a pH of 2.5. It was concluded that the higher crosslinking degree due to the composite structure results in high swelling properties that facilitate diffusion of the crosslinker ions to the rest of the polymer—facilitating its production. Furthermore, higher concentrations of pentasodium tripolyphosphate resulted in lower swelling ratios due to increased nodes created during crosslinking, which result in slower water penetration. However, this specific mixture did not noticeably increase the bulk membrane density or the crystalline size [56].

Another composite can be created by introducing negatively charged sulfate ions into a wetted chitosan film, which results in ionic crosslinking that makes the hydrogel insoluble in water. This produces a polymer well suited to electrode binders due to being stable upon heating and under electric loading, with applications for batteries and transmitters [57]. Metal ions were later introduced in another study that demonstrated that the chitosan structure has a strong affinity to copper ions. The copper (0.1 M copper ion from copper sulfate) and chitosan (2% acetic acid solution with 0.5 wt% chitosan) solutions were mixed at room temperature where a solid precipitate was created from the ionic bonding. The crosslinking effect of the copper ions decreased the solubility of chitosan and resulted in the solution becoming cloudy as it precipitated. Multilayered structures were created and described as the spontaneous transition from oriented fibers to multiple layers. One notable feature of this method is that when analyzed with X-ray diffraction, no copper diffraction peaks were identified. The copper ions were able to change and improve the structure without directly being included in it and reduced the toxicity of the polymer [58]. This is a notable feature of the composite hydrogel, which reduces the need for further purification processes.

To improve the crosslinked properties, foaming with carbon dioxide is performed, which is followed by chemical initiation using ammonium persulfate and tetraacetylethylenediamine, which results in a super-porous chitosan network. Chitosan concentrations below 50% *w*/*v* were effective, making the process comparable to traditional chemical crosslinking methods. Macropores formed by entrapped bubbles facilitated stabilization of the porous structure during crosslinking. Mechanical properties are increased sufficiently to withstand intragastric pressures [59].

Synthetic polymers such as diepoxy PEG can be used in place of natural polymers or chemicals. This is a derivative of polyethylene glycol, and it exhibits high hydrophilicity and increases the density of the polymer with increasing weight fractions of diepoxy PEG [60]. This synthetic polymer is created by combining a PEG molecular weight of 2000 g/mol and composing 30% *w*/*v* with a chitosan/acetic acid (0.4% *w*/*v*) solution. This is cast in a mold, washed for 24 h to remove the acetic acid, and freeze-dried for 48 h to obtain the dry weight. Furthermore, lysozyme (1 mg/mL) was later added and set for 24 h to determine the surface effects. It was found to degrade the surface and leave behind a rough patch. Weight loss was used to determine the degradation rate and was found to lose over 21% of the chitosan’s hydrogel total mass over 32 days. Higher concentrations of PEG, such as 44% *w*/*v*, reduced the degradation to at most 5% over the same time. This allows the structure to be altered for its intended purpose, with smoother surfaces having improved lubrication and lower friction while higher roughness surfaces can increase the adhesion and potential for wear. The addition of synthetic polymers contributes to the potential use cases of each hydrogel by altering the properties, such as degradation time, depending on the use case.

The choice of monomers and crosslinking chemicals used is vital in determining the properties and under what conditions the polymer will swell. One of the most common polymers is Poly(N-isopropylacrylamide) which is created from free radical polymerization [61]. Other mixtures are sensitive to hydration changes, where the shape-morphing properties of the polymer can be exploited to create hollow channels for replicating vascular tissue. The combination of various materials and chemicals in a single composite hydrogel provides variable mechanical structures for different vascular tissues. Diameters as small as 20 μm can be made with this method and combining the sensitivity with composite polymers causes complex structures to be made by the differing deformations of different parts of the total [62]. A summary of crosslinking methods can be found in Table 2.

## 3. Drug Delivery and Biocompatibiltiy

### 3.1. Chitosan Hydrogels for Drug Delivery

Chitosan hydrogels have been found to form effective, injectable carriers for localized drug delivery within venous systems. These hydrogels can encapsulate therapeutic agents, such as anti-inflammatory drugs, anticoagulants, or thrombolytic agents. Controlled release at the target site is possible through modification of the hydrogel’s structure and reduces the need for invasive surgery. PH-sensitive hydrogels have been shown to effectively target specific sites with over 95% entrapment efficiency and provide short- or long-term drug release [63]. Diclofenac sodium powder (50 mg), a non-steroidal anti-inflammatory drug, was dissolved together with a solution of 0.6 g of chitosan, 2% formic acid, and 50 mL of distilled water at 50 °C over four hours [64]. A linear relationship between time and drug release percentage was discovered after in vitro testing, with 90% drug release occurring within 130 min of injection. Furthermore, the drug encapsulation efficiency, a measure of the fraction of the drug loaded in the hydrogel that is present in the polymer network, increased from 64 to 84%.

**Figure 3:**
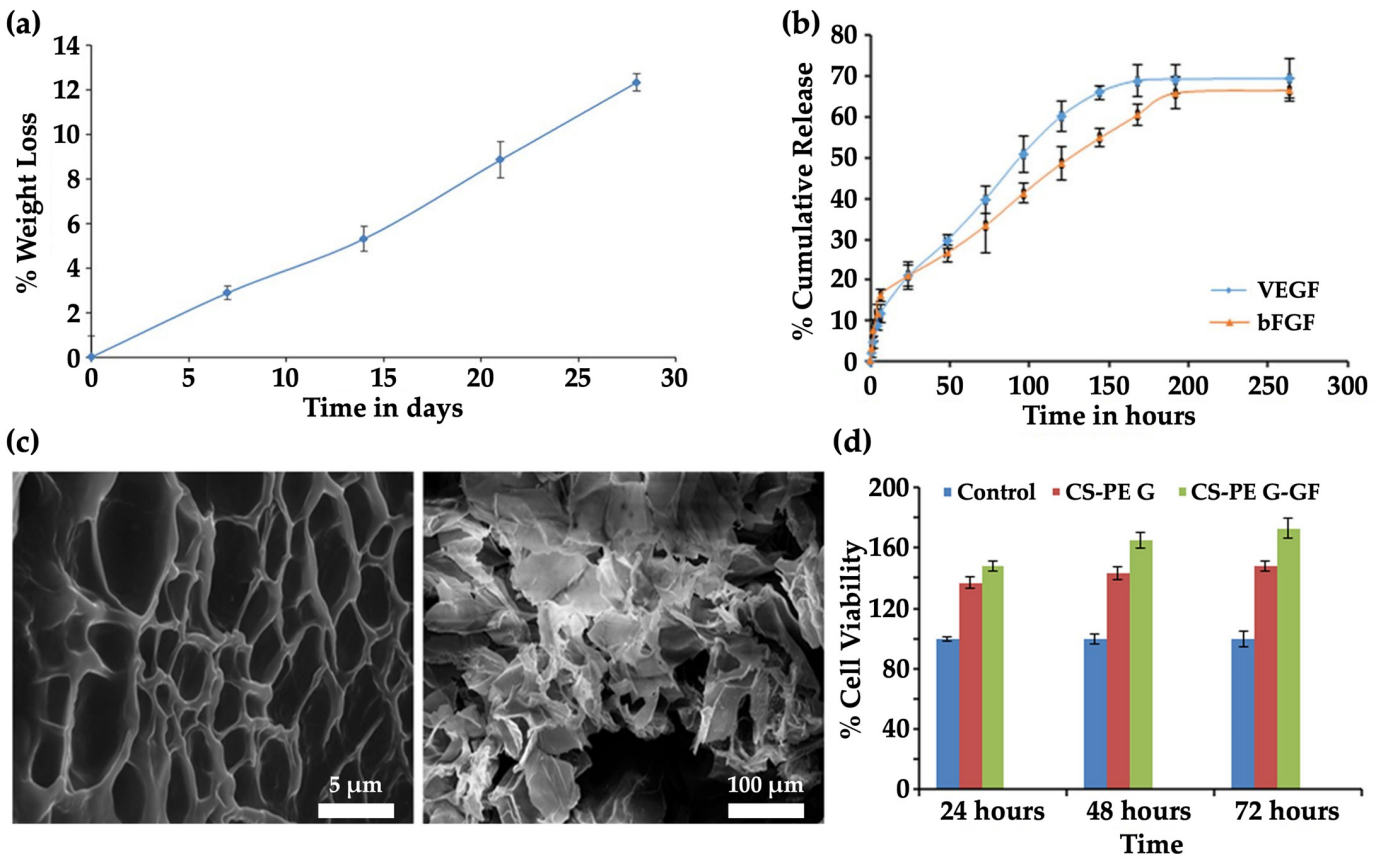
Fabrication and characterizations of bFGF-loaded chitosan hydrogels. (**a**) Weight loss behaviors over time for selected hydrogel. (**b**) Cumulative bFGF releases from chitosan hydrogels over time. (**c**) Overall structure represented by SEM images of various synthesized scaffolds. (**d**) In vitro cell viability of selected hydrogels. Figures were reproduced for quality from an open access article [65], distributed under the Creative Commons Attribution License.

Basic fibroblast growth factors (bFGFs) were incorporated into the chitosan hydrogel structure which was composed of chitosan dissolved in formaldehyde [65]. After mixing with polyethylene glycol as a linker, bFGF and heparin were added to the solution. The bFGF is a naturally occurring protein and has increased in medical usage due to its vital role in various cellular processes—especially angiogenesis, the creation of new blood vessels. During an in vitro hemolysis test, bFGF contributed to angiogenesis by extending release to 60% over 250 h. Improved vascular complexity was observed with an increase in cell viability of blood vessels from 120% to 160% as shown in Figure 3. Existing damage to blood cells reached negligible activity (less than five percent occurrence) after ten days.

Chitosan hydrogels used for promoting angiogenesis can be introduced directly to surgical sites for direct and targeted applications. One such application is for the treatment of strokes with chitosan hydrogels carrying VEGF, a vascular endothelial growth factor that is crucial in the formation of new blood vessels [66]. The hydrogel was produced from 0.5 g of chitosan dissolved in 40 mL of lactic acid and 1.5 g of succinic anhydride was added and stirred for 24 h. After modifying the pH to between 6 and 7, the succinyl-modified chitosan was precipitated, filtered, dissolved in distilled water, and stored at 4 °C. Finally, a sodium periodate solution was added and agitated at room temperature for two hours in the dark, stirred for an hour, then freeze-dried. After dissolving in a phosphate-buffered solution to 20 mg/mL, the crosslinked hydrogel was produced and coated with 50 ng/mL of VEGF. The delivery method was tested in vivo by application directly to the peri-ischemic region of a rat’s brain, the area immediately surrounding an area of insufficient blood flow in the brain, over seven to fourteen days. Motor function was analyzed and compared with a control group to determine VEGF effectiveness with hand preference falling from 55% to 22% and grip force improving from 500 g to 700 g—displaying marked improvement in motor function and post-stroke healing.

While anticoagulants are useful for preventing strokes, thrombosis, and related blockages to vascular pathways, embolization, or for the purposeful blocking of blood flow, they can also be used to stop bleeding and devascularize organs. One such compound is doxycycline, which is used extensively for preventing abdominal aortic aneurysms, which can result in a fatal rupture and internal bleeding [67]. The chitosan solution was prepared with 3.33% *w*/*v* chitosan mixed with a 0.1 M HCl solution at room temperature. Sodium bicarbonate was used as the gelling agent, and doxycycline was mixed in the syringe immediately before use. A two-stage profile of the doxycycline’s release (0.1% *w*/*v*) was observed in vivo, with a rapid burst release during the first 24 h followed by a slow, continuous release for up to 168 h. Since the chitosan hydrogel could be delivered by catheter, a minimally intrusive operation was needed.

**Figure 4:**
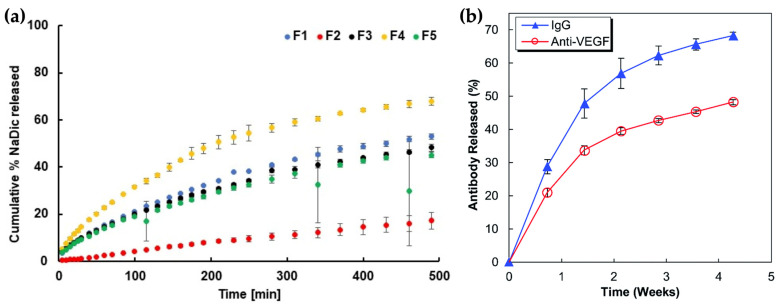
(**a**) Cumulative release profiles of diclofenac sodium from inflammation-targeted hydrogels in hyaluronic acid (HA) solution (pH = 8.20, 5.71, 7.87, 7.71, 7.69). The figure was obtained from an open access article [68], distributed under the Creative Commons Attribution License. (**b**) Cumulative releases of antibody (non-specific IgG or anti-VEGF) loaded in alginate–chitosan polyelectrolyte complexes over 30 days. The figure was reproduced or adapted from [69], with permission from Royal Society of Chemistry, 2026.

PH-sensitive hydrogels have been shown to effectively target specific sites and provide short- or long-term drug release. Diclofenac sodium powder (2 g), a non-steroidal anti-inflammatory drug, was dissolved together with a solution of chitosan, hyaluronic acid, and polyacrylic acid [68]. A linear relationship between time and drug release percentage was discovered after in vitro testing, with 50% drug release occurring within 500 min of injection as shown in Figure 4a.

In a similar fashion, therapeutic antibodies can be delivered intravenously but this system of delivery requires large doses to ensure that enough therapeutic antibody reaches the target site [69]. This can lead to challenges when variable dosing is needed. A 1% *w*/*v* solution of alginate was mixed with a 13% *w*/*v* solution of chitosan before adding bevacizumab—a medication that inhibits tumor growth by targeting the vascular endothelial growth factors. This bevacizumab-carrying hydrogels were stored for thirty days to determine the shelf life, and it was discovered that this growth factor is anti-VEGF and inhibits endothelial cell functions and provides sustained delivery. The hydrogel effectively delivered bevacizumab during an in vitro test with approximately 30% delivered in one week and 65% delivered within four weeks as shown in Figure 4b.

The delivery of siRNA, an essential gene therapy drug that assists in degrading specific mRNA molecules, has been shown to reduce the progression and effects of tissue factors that thicken vascular smooth muscle cells and promote a series of cardiovascular diseases such as hypertension and acute coronary syndrome [70]. The hydrogel was prepared with a chitosan of 130–160 kDa and degree of deacetylation of 86% along with a hydroxybutyl solution of 1 mg/mL. After mixing with 0.2 M acetic acid and a 0.5 mg/mL tripolyphosphate solution to a concentration of 20 µmol/mL, 1 mL of SiRNA nanoparticles was added and stirred for 30 min. During the in vitro study, cell apoptotic rates, or cell death rates, of the targeted excess vascular muscle cells increased from between 5.1% and 7.73% up to 42.25% after 72 h post-transfection. The siRNA was delivered at a rate of 74% which demonstrates the hydrogel’s advantage in attaching to vascular tissue.

### 3.2. Biocompatibilities of Chitosan Hydrogels

Chitosan hydrogels can be modified during fabrication to optimize for vascular tissue healing and developing scaffolds for vascular tissue growth. High porosity and angiogenesis enhancement (repair from existing vascular tissue) are key properties of the scaffolds formed by the hydrogels which form the base frame material to support further tissue formation [71]. Analysis of chitosan hydrogels produced from β-chitin displayed cytocompatibility, the ability to operate without damage to cells, and were studied using HDF and NIH 3T3 cells. Both cell types are commonly used in biomedical research as replacements of human tissue and showed strong compatibility with the hydrogels [72].

3T3 mouse fibroblasts are a widely used cell line derived from Swiss mouse embryos and are commonly used for fat cell development and studies of connected vascular conditions such as glucose, insulin, and angiogenesis [73]. First, 0.1 g of high-molecular-weight (300 kDa) chitosan was dissolved in 10 mL of CH_3_COOH solution in water for 24 h. After centrifuging at 1000 rpm for 15 min, the hydrogel was produced. Cells were seeded during the in vitro test at densities of 5 × 10^4^ cells/well in a 48-well culture plate to analyze biocompatibility. No significant (*p* < 0.05) cell damage or toxicity was observed, and cell proliferation instead increased the number of viable cells to 31% higher within 24 h and 50% higher within 48 h. Human cells were cultured and tested which resulted in cell proliferation increasing 38% and 40% within the same time periods.

**Figure 5:**
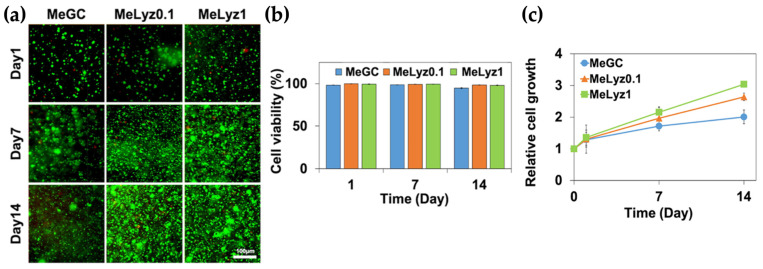
Methacrylated glycol chitosan hydrogels showing (**a**) live/dead staining (green/red) images of the BMSCs over 14 days, (**b**) quantified cell viability, and (**c**) relative cell growth of the BMSCs. Figures were reproduced or adapted from [74], with permission from ACS Publications, 2026.

Another method of improving biocompatibility involves methacrylated glycol chitosan hydrogels, which were prepared with 2% *w*/*v* glycol chitosan mixed with distilled water and glycidyl methacrylate at a molar ratio of 1:1 [74]. After 40 h, the solution was adjusted to a pH of 7.0, dialyzed with distilled water for 16 h, lyophilized and rehydrated with a phosphate-buffered saline to obtain a 2% *w*/*v* methacrylated glycol chitosan. After photo-crosslinking, the hydrogel was exposed to lysozyme to analyze its effectiveness as a degradable tissue scaffold. With the hydrogel degrading between 10% and 70% depending on the lysozyme concentration (0.1 to 10 mg/mL respectively), any potential long-term toxicity can be mitigated by purposeful degradation of the hydrogel. Bone mesenchymal stem cells, which were differentiated into vascular endothelial cells to repair vascular tissue, were used to test in vitro cell viability as shown in Figure 5. Cell viability remained at 95% or above over 14 days and cell growth improved by a factor of three as shown in Figure 5.

Gas foaming is another method to improve the biocompatibility of chitosan [75]. Prior to beginning the process, solubility data of the gas, such as CO_2_, is collected to determine the ideal temperature and pressure to inject the gas at. This minimizes exposure time of cells to CO_2_, since after about five minutes the cells begin to lose viability. Another option would be to replace the pores with human cells during the foaming process simultaneously. Furthermore, the supercritical CO_2_ used can also sterilize the solution. The hydrogel allowed strong cell attachment and colonization of fibroblast cells, a major cell type in the outer layer of blood vessels, and was able to penetrate the structure due to the pores opened by the gas foaming. Cells became spindle-shaped after four days with new cell penetration reaching up to 200 µm, while also introducing oxygen into the vascular tissue. Cell viability increased from 78% on day one to 90% on day seven during the in vitro study.

**Figure 6:**
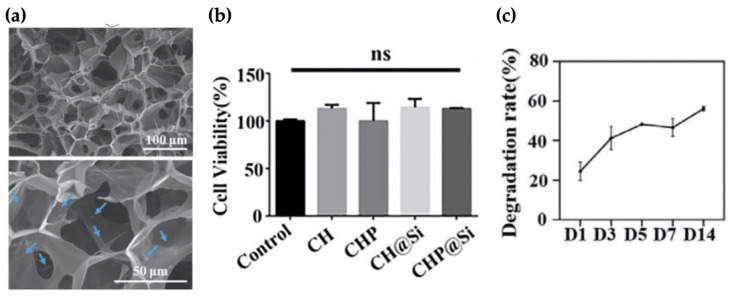
Composite chitosan and silicon hydrogels, showing (**a**) SEM images of composite chitosan hydrogels crosslinked from β-glycerol phosphate disodium salt pentahydrate and hydroxyethyl cellulose, where blue arrows indicate the tightly bound mesoporous silica nanoparticles on porous chitosan structure, (**b**) cell viability over 24 h, and (**c**) hydrogel degradation rate over 14 days. Figures were obtained from an open access article [76], distributed under the Creative Commons Attribution License.

Another method of improving biocompatibility is using composite chitosan hydrogels, which were produced through crosslinking 200 mg of low-to-medium-viscosity chitosan with 0.5 mL β-glycerol phosphate disodium salt pentahydrate and 0.5 mL of hydroxyethyl cellulose [76]. The composite hydrogel was tested in vitro by application to human umbilical vein endothelial cells to determine biocompatibility. The hydrogel was applied to a 96-well plate of endothelial cells at 37 °C for 24 h under hypoxic conditions to simulate damaged vascular tissue. As shown in Figure 6, cell viability improved to 110% of the control group within 24 h. Furthermore, the composite hydrogel exhibited a pH of 7.0 and reduced the expression of pro-inflammatory factors in the cells.

Another composite hydrogel is produced with polyvinyl alcohol and chitosan and tested on Petri dishes containing bovine aortic smooth muscle cells to evaluate cell compatibility for future applications in vascular diseases [77]. The hydrogel was formed with a 10% polyvinyl alcohol solution and combined with a chitosan hydrogel prepared from 0.2 M acetic acid and between 0.5% and 2.0% *w*/*v* of 810,000 g/mol chitosan. The two were blended at a 3:2 ratio and cast into a 0.5 mm by 34 mm mold and crosslinked by freezing at −20 °C for 12 h and thawed for 12 h. Each hydrogel mold had 1 mL of aortic smooth muscle cells applied during the in vitro test which contained 13,000 cells and was grown for 24 to 96 h before analysis. Cell viability increased by 10%, with the 1.0% *w*/*v* chitosan hydrogel displaying the largest increase in cell growth. Furthermore, the percentage of cells undergoing apoptosis, or programmed cell death, decreased by 6% after five days.

These hydrogel combinations can be injected into tissue as a minimally invasive method of application and can be modified to be liquid at room temperature while gelling rapidly once they enter the body—eliminating the need for surgery [78]. Losses in medicine effectiveness often decrease with increasing distance from the target area they are applied to, and injections can be guided by physicians to the exact area of concern. Chitosan hydrogels crosslinked with low-molecular-mass hyaluronic acid (20–200 µg HA/mL) were shown to increase endothelial cell migration by 30% when put into contact with bovine aortic cells [79]. Furthermore, the quantity of aortic endothelial cells increased 120% in comparison to the control group. Molecular masses of over 800 µg HA/mL were tested to determine if cell toxicity was present, and no side effects were found other than returning cell proliferation to the levels of the control group.

Graphene oxide was introduced in 2023 with a new method of reducing its toxicity. Normally, only very small doses are viable for human use, but the addition of chitosan reduces the toxicity by lowering its solubility and reducing cell damage [80]. Chitosan forms the basis of the polymer while the graphene oxide is used to modify the response [81]. The hydrogel was produced by a combination of 3% *w*/*v* chitosan and 5% *w*/*v* polyvinyl pyrrolidone, stirring the solution for 24 h, after which a solution of 5% *w*/*v* polyethylene oxide was added. Finally, the hydrogel was finished after electrospinning at 0.3 mL/h with the graphene oxide. A concentration of 1.5% graphene oxide resulted in 40% greater mesenchymal stem cell viability in an in vitro test after 72 h. These stem cells play an important role in promoting angiogenesis through the release of VEGF and treating diseases such as infarctions, and graphene oxide and chitosan hydrogels demonstrated no notable toxicity above 1% concentration.

**Figure 7:**
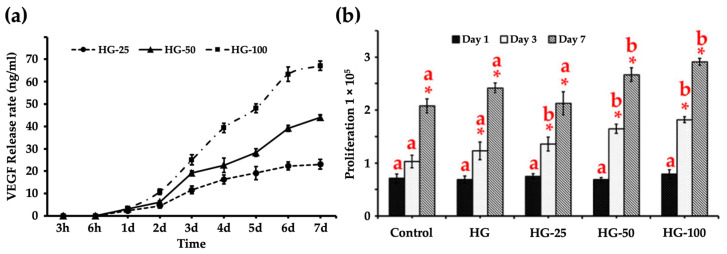
(**a**) Cumulative release profiles of VEGF from chitosan hydrogels. (**b**) Increase in cell proliferation during in vitro trial after application of chitosan hydrogel. * and different alphabets denote statistical significance, * vs. day 1 and different alphabets vs. control group, respectively, *p* < 0.05. Figures were reproduced for quality from an open access article [82], distributed under the Creative Commons Attribution License.

Further improvements in biocompatibility of chitosan hydrogels are demonstrated by improving neovascularization of vascular endothelial cells with a photo-crosslinkable chitosan hydrogel as shown in Figure 7 [82]. Created with a 1% *w*/*v* chitosan solution, crosslinked with collagen in an acetic acid solution, and loaded with between 25 and 100 ng/mL of vascular endothelial growth factors (VEGF), the hydrogel was cultured with cells and improved cell proliferation from VEGF treatment during the in vitro trial. Cell proliferation was determined by staining between one and three weeks of treatment with an increase from 2 × 10^5^ cells to 3 × 10^6^ cells, drastically increasing the blood flow to the region.

## 4. Vascular Tissue Engineering and Stent Applications

### 4.1. Chitosan Hydrogels for Vascular Tissue Engineering Applications

**Figure 8:**
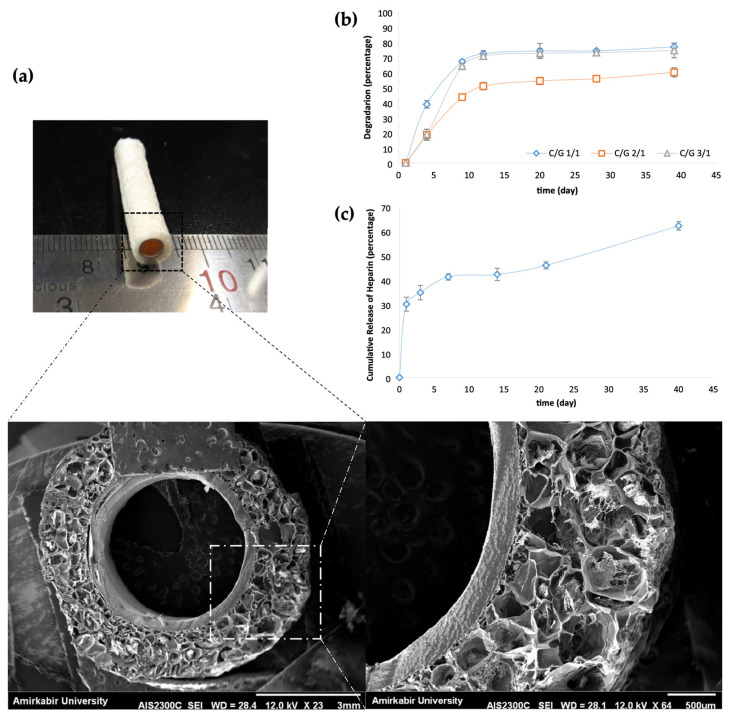
(**a**) Photos of the tubular scaffolds electrospun from PU/gelatin-heparin microfibers. (**b**) The weight loss of crosslinked gelatin-heparin fibrous scaffolds at various concentrations in vitro for 40 days. (**c**) The heparin release curve of gelatin-heparin fibrous scaffolds in PBS at 37 °C (heparin, 1 wt%). Figures were obtained from an open access article [86], distributed under the Creative Commons Attribution License.

Chitosan hydrogels used for vascular engineering have been shown to meet the basic requirements of biocompatibility and mechanical and surface properties [83]. Biological coatings are an important surface modification applied to chitosan hydrogels to adjust the interaction and improve blood cell compatibility [84]. PEG was covalently grafted onto the hydrogel through UV photopolymerization with grafting densities between 400 and 1000 g/mol [85]. The repair of blood vessels is accelerated by the hydrogel’s ability to increase platelet adhesion by 500 per mm^2^ and is crucial in maintaining the stability of vascular tissue during treatment. Electrospun fibers made from a heparinized polyurethane thermoplastic, earlier used for improving biocompatibility, as shown in Figure 8, can also be used to provide a surface for improving endothelization in the first few days of a graft and can also be used to create completely artificial vessels [86].

One composite chitosan hydrogel introduced incorporated copper, tin, and nickel sulfides [87]. The copper ions are recognized as an effective antimicrobial agent and promote angiogenesis—the process of forming new blood vessels from pre-existing ones. The copper ions utilize the same HIF-1α pathways used by hypoxia and activate a similar gene response which promotes angiogenesis [88]. During an in vitro trial, VEGF increased between 2.06 and 3.62 times over the control group, which consisted of a chitosan hydrogel without Cu, Tn, or NiSO_3_. After fourteen days, the VEGF expression remained at 1.99 times the control. With the VEGF, a higher degree of neovascularization occurs, and enhanced angiogenesis supplied additional oxygen and nutrients to nearby vascular tissue and assisted in propagating the vessel growth. Furthermore, 99% of bacteria was eliminated within four hours of contact and prevented inflammation from interfering with the vessel growth.

Alginate mixed with the chitosan hydrogel forms a reactive mixture at a pH level of 4, which is the typical value that vascular tissue begins to heal when damaged [89]. The hydrogel was applied to endothelial cells during an in vitro trial over a period of eight days in which the number of vein junctions improved from 35 to 95, and the total branching length due to angiogenesis increased by a factor of 2.5. Good hemocompatibility was demonstrated with cell viability remaining over 94.3% throughout the treatment, and damaged tissue was reduced by 12%. The repair and proliferation of vascular tissue were greatly improved by the angiogenesis induced by the hydrogel as a result of improved cell migration without negative effects or excessive cell damage.

Hydrogels are also effective in repairing venous malformations that create low-flow or cavitary structures. This method uses CO_2_ foaming with the addition of sodium tetradecyl sulfate, which can easily be washed away if needed. Using only hydrogels, both weight fractions used were more effective than 3% sodium tetradecyl sulfate. This was verified by using mice with lesions which were treated to determine whether ulcers were developed or plugs in the veins were destroyed—indicating that the combination is too toxic [90]. The challenge of cell toxicity was addressed through the introduction of induced pluripotent stem cells, where the DNA of the somatic cells were reprogrammed to become pluripotency cells, which can become any type of cell. The genetic makeup of the DNA can be altered to correct disease-causing mutations or add special modifications. However, concerns have emerged about this method because of the possibility for chromosome changes that can replicate and cause cancer or other diseases. Endothelial cells and smooth muscle cells can be harvested from the patient instead but require a more invasive blood vessel biopsy and typically generate low quantities of vessels.

Instead of directly promoting the generation of cells, vascular grafts were developed to provide a strong structure for vein development [91]. As a trial, endothelial cells were obtained from a sheep’s carotid artery and seeded for 21 to 28 days in vitro to produce a dish with only that structure. The burst strength was observed to increase to more than 300 mmHg. This is suitable for most applications except for larger veins, arteries, or the heart that require burst pressures up to 300 mmHg. Furthermore, the wall thickness of the vascular grafts increased from 0.50 mm to 0.73 mm, and the suture retention improved from 12.0 to 64.3 g after 28 days. These vascular grafts were integrated into the existing vascular tissue and demonstrated improved mechanical stability after a three-week trial and provided the base material for vessel growth. Furthermore, a more structured and layered process of tissue formation was observed which resulted in increased treatment effectiveness.

Other mechanical properties can be considered for designing vein grafts include the effect of the pulsating pressure and how the diameter changes along with it. Non-degradable polymers are typically need a combination of other chemicals with the base chitosan hydrogel due to its inherently weak structure. On the other hand, decellularization of native blood vessels, otherwise known as taking apart vessels to their base parts, is possible and can address concerns of adverse immune responses [92]. Long-term research is needed in determining the possible side effects of the vein grafts. One clinical study tested a poly-L-lactide acid hydrogel in 25 children which were created with bone marrow and mononuclear cells [93]. However, over 11 years, seven of the patients experienced narrowing of the blood vessels without any noticeable side effects. However, this could potentially cause issues or necessitate another surgery later in life.

When developing artificial blood vessel grafts using chitosan hydrogels, pulsating flow is typically induced to mimic the activity of natural blood vessels [94]; 50 to 60 mmHg cyclic loading is applied to the chitosan hydrogel and improved compliance from 6.8% to 11.5%, which is a measure of the vessel’s ability to expand and return to its original size during pulsating flow. Maximum burst pressures were between 840 and 1313 mmHg; while underneath the body’s typical 2134 mmHg, this means that the hydrogels are applicable for smaller coronary arteries. Increasing weight percentages from 5% to 10% leads to a doubling of suture strength to 110 g of force and proved adequate for sealing native vessels during grafting. Oxygen and nutrients are typically supplied to the polymer before implantation to prevent absorption of the body’s cell nutrients and led to a vessel graft prepared and conditioned with in vitro characteristics.

### 4.2. Chitosan Hydrogels for Vascular Stent Applications

Venous stents are a method of inserting a mesh tube into a vein to keep it open and allow for normal blood flow. In disease or trauma cases involving the narrowing of veins, repairing the vessel can be considered rather than completely replacing the vessels. By taking a chitosan hydrogel and impacting copper ions, it is possible to use its stimulus-driven properties to be liquid when inserted into the vein, and then when an electrical signal of between 0.5 and 2 amps/m^2^ is applied, the hydrogel will solidify and deposit as a coating onto a metallic stent 160 μm thick [95]. Catechol groups are used to strongly bind the two polymers together with the copper ions and improve stress properties by 1 MPa at a strain of 4.7% without permanent deformation. The increased stress capacity enabled the stents to dissipate stress more effectively under blood flow, and endothelial cell density, measured by optical density, rose from 0.05 to 0.42 in the in vitro study—indicating improved cell proliferation of the new vascular tissue. Furthermore, the hydrogel can prevent the formation of biofilms in inhibitory zones of 34 to 32 mm and avoid future buildup in the same vein and contributes to the cell proliferation [96]. However, buildup can still occur at another location upstream or downstream of the stent. Vitamin E was employed within the hydrogel to form a resistant coating to extend the lifetime of the device while not affecting its properties or function [97]. Biofilms with the addition of Vitamin E were reduced by up to 96.6% due to its antiadherent properties which prevented bacteria adhesion and reduced the possibility of restenosis or the narrowing of the artery after a stenting procedure.

**Figure 9:**
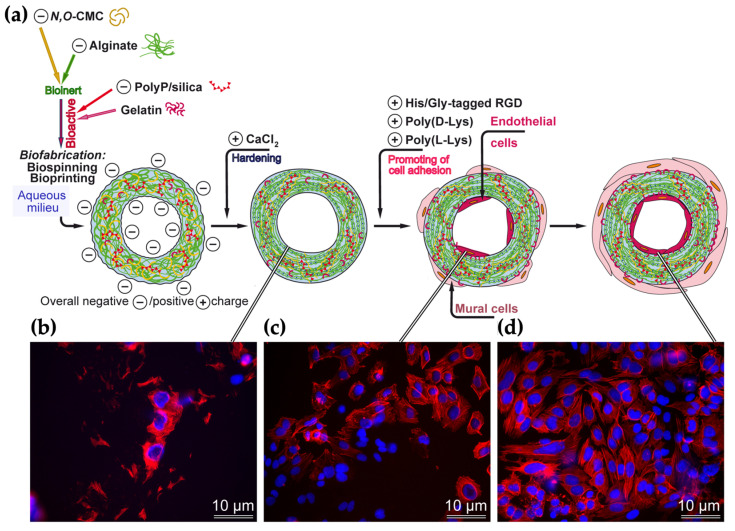
(**a**) Fabrication of biomimetic tissue-engineered blood vessels using negatively charged *N*,*O*-carboxymethyl chitosan and alginate. Fluorescent images of (**b**) basic scaffold alone, (**c**) basic scaffold, supplemented with poly(d-Lys), and (**d**) basic scaffold, supplemented with His/Gly-tagged RGD. Figures were obtained from an open access article [98], distributed under the Creative Commons Attribution License.

Smaller diameter stents have presented difficulties in effective treatment due to the challenges in delivering them and narrowing of the arteries. To address this challenge, alginate and gelatin were set in a fixed sequence with a *N*,*O*-carboxymethyl chitosan, silica, and polyphosphate scaffold as shown in Figure 9 [98]. Scaffolds as small as 0.8 mm inner diameter were successfully created since the scaffold shrinks during hardening, and are smaller than the average human muscular artery wall. Scaffolds developed with this method lasted up to four weeks with comparable burst pressures and increased the cells per square millimeter by over a factor of nine, from 25 to 310 cells/mm^2^. The scaffold was left to cultivate endothelial cells for two weeks with both a basic scaffold and the new hydrogel. After the two-week period, the cell proliferation rose with the chitosan hydrogel and fully populated the matrix as seen in Figure 7. The risk of thrombosis after the procedure is minimized due to the inert scaffold and the elastic modulus (100–1400 kPa) can be adjusted to match the surrounding tissue.

Even with strong and well-placed stents, intimal hyperplasia, a condition where the innermost layer of the blood vessel thickens and narrows the vessel, is a major concern for any vascular scaffold. This process is akin to scar tissue building and results in restricted blood flow, higher thrombosis risk, and graft failures. The need for medical intervention in either removing or repairing the stent adds additional stress and risk to the patient [99]. The risk is reduced when preventative drugs are delivered to the interior of the stent such as rapamycin, a drug commonly used to prevent organ rejection after transplants. The hydrogels can provide effective, local applications of anti-inflammatories and promote vessel growth while also being able to compensate for associated risks in a single package. Polycaprolactone was added to the gelatin/hydrogel structure further reduce the possibility of hyperplasia and provided continuous support of endothelialization, the growth of vessel-lining cells that mimic the natural vessel structure in the repair area [100].

### 4.3. Chitosan Hydrogels in Clinical Applications

Chitosan hydrogels are oftentimes used in support of medical procedures such as in endoscopic sinus surgery to accelerate healing and improve final outcomes, by assisting in vascular growth. A clinical trial of 26 patients was conducted to see the hydrogel’s effectiveness and the gel was applied randomly to a single nasal passage to assess the treatment of the same patients [101]. The patients were from 18 to 79 years old with a 12:14 male-to-female ratio. The chitosan hydrogel was observed to increase the rate of epithelialization of the blood vessels and partially bypass the inflammation to heal the vessels deeper in the nasal passage that were otherwise blocked from direct contact. Ostial measurements were taken at 2, 8, and 12 weeks post-operation to assess how open the nasal passages remained. The maxillary ostia’s outcome was significantly impacted with the rate of second procedures dropping from 47% to 27% with the change in method. After 12 weeks, the average ostial area open ranged from 2.6 to 36.6 square millimeters more than the control of no gel use. Throughout this study, the chitosan hydrogel contributed to surgery improvements via increased epithelialization and vascular tissue regeneration, which highlight the hydrogel’s ability to support angiogenesis.

**Figure 10:**
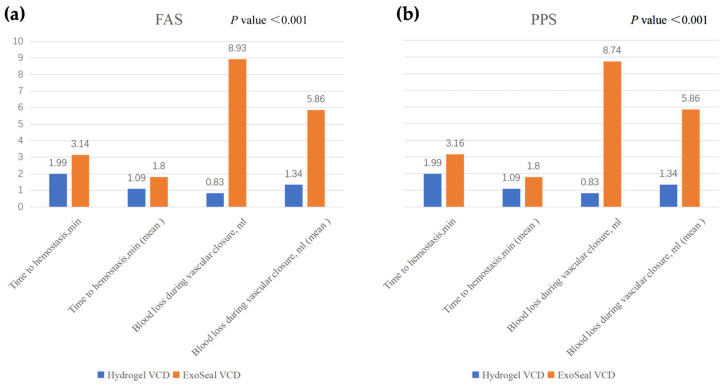
Time to hemostasis and blood loss volume during vessel closure. (**a**) FAS, full analysis set; (**b**) PPS, per-protocol set; VCD, vascular closure device. Figures were obtained from an open access article [102], distributed under the Creative Commons Attribution License.

In August of 2023, a clinical trial for chitosan hydrogels was performed to determine the viability of using the structures as a vascular closure device to achieve hemostasis of the small hole in the artery created by catheterization as shown in Figure 10 [102]. The objective was to minimize the material absorption times and the blood loss produced during deployment. To gain the best results, a group of 202 patients between the ages of 18 and 80 were randomly selected without consideration of sex who were undergoing the procedure. Patients with previous surgical experiences, with calcification at the area, or when the puncture was lower than the lowermost edge of the inferior abdominal artery wall were excluded; 100 of the patients were in the control group while 102 patients experienced the novel treatment, with 99.02% experiencing positive effects from the hydrogel with fewer complications. The control only experienced 94.00% effectiveness. The chitosan hydrogel lowered blood loss by 8.10 mL on average and 1.17 min faster to stop bleeding. Figure 8 shows the time to hemostasis of the hydrogel compared with the control ExoSeal Vascular Closure Device during both the full analysis set and per-protocol set. The chitosan hydrogel improved clinical results through greater biocompatibility and minimizing associated risks due to prolonged endothelial support. Furthermore, the risk of iatrogenic vascular injuries, which are unintentionally caused during medical interventions, are minimized due to the hydrogel providing a hydrated and favorable environment for vascular repair. Finally, no device defects were found in the hydrogel treatment while two incidents occurred with the competitor brand Exoseal vascular closure device.

A separate clinical trial addressing the same issue was completed during the same year which addresses the exterior bleeding control of the puncture site and damage to associated vascular tissue within the body [103]. A chitosan hydrogel-based hemostatic pad was used for the trial of 204 patients, with half of those being the control group. Patients above the ages of 18 undergoing elective angiography were eligible while those with coagulation disorders, pregnancy, or myocardial infections were excluded. Ecchymosis, the accumulation of blood under the skin due to trauma was used as a primary method of determining treatment effectiveness. The rate of ecchymosis was decreased by 12% in treated patients, along with two hours less rest time required, and zero occurrences of pseudoaneurysm. Time to hemostasis was decreased by 50.33% from the manual compression method.

Despite promising clinical outcomes in vascular engineering applications, chitosan-based hydrogels have seen limited translation into approved vascular medical products and procedures. As highlighted in clinical studies, most chitosan-hydrogel based studies remain in early-phase trials or lack extensive long-term follow-up data. Although these hydrogels have demonstrated strong preclinical potential, they still lack the extensive clinical validation required for regulatory approval. One key limitation identified across clinical studies is the variability in chitosan sourcing and quality, particularly in molecular weight and degree of deacetylation, both of which can significantly influence material performance, degradation, and reproducibility. In addition, manufacturing remains a critical challenge, where scalability is constrained by difficulties in consistent production, standardization, and the potential introduction of allergens from contamination [104]. After fabrication, chitosan hydrogels may exhibit limited storage stability due to the moisture sensitivity and are often designed to undergo time-dependent degradation, which can constrain shelf life. Furthermore, common industrial sterilization methods may alter the material properties of chitosan hydrogels, since UV light, autoclaving, and gamma radiation can induce structural changes that affect mechanical strength and performance.

## 5. Conclusions and Future Works

Chitosan hydrogels are versatile biomaterials that address the growing clinical needs for highly customizable and patient-specific treatment options. Introduction of growth factors and minerals improve the healing rates of the generation and viability of new vascular tissues. Furthermore, the tunable properties of the hydrogels enable the development of patient-specific therapeutic systems. Self-assembling scaffolds and coatings improve quality of life and lower the risk of blocked veins in patients. With the incorporation of biomaterials, biocompatibility is improved as demonstrated through laboratory and clinical trials.

Despite these developments, several challenges must be addressed before widespread clinical translation can be achieved. Improving the mechanical strength of chitosan hydrogels remains essential for applications requiring prolonged drug delivery or structural support in larger vascular tissues. In addition, greater control over hydrogel degradation is needed to ensure that it aligns with therapeutic timelines. Further clinical studies are required to determine the long-term effects of chitosan hydrogels including hemocompatibility, thrombosis risk and other adverse effects.

Future research should focus on developing scalable and cost-effective fabrication methods, as most current studies remain limited to laboratory-scale production. Consistent quality and performance must be established to identify optimal blends for patient use and sterilization methods. Furthermore, standardized databases documenting hydrogel formulations and processing conditions may facilitate regulatory approval. Although encouraging preclinical and clinical results have been reported, larger-scale and long-term clinical studies are required to validate performance. Overall, chitosan hydrogels provide a promising platform for drug delivery and vascular repair, and continued advances in material design and fabrication are expected to support their future clinical adoption.

## Figures and Tables

**Table 1:** Chitosan hydrogel chemical crosslinking properties [38].

-OH Groups	-NH_2_ Groups	Flexibility of Matrix
Enhance hydrophilicity which absorbs water	Cause protonation in water with acidic mediums	Controlled crosslinking density
Controlled swelling for drug delivery	pH-sensitive properties for targeted drug delivery	Tuning of hydrogel with blended polymers
Ability to include metal ions for bone tissues	Enhanced cell adhesion	Mechanical strength from hydrogen and ionic bonds

**Table 2:** Comparison of crosslinking strategies for chitosan hydrogels.

Crosslinking Method	Mechanical Properties	Cytotoxicity	Degradation	Vascular Suitability
Chemical	High strength and tunable stiffness	Low–moderate from remnant chemicals	Slow rate of degradation	Strong networking and scaffolds
Physical	Moderate strength and tunable stiffness	Low–moderate with natural materials	Slow rate of degradation	Biocompatible for low-strength applications
Stimulus-Responsive	Variable strength with thermal stability	Low with minimal chemical use	Variable as light stimulus can cleave bonds	Smart drug release systems
Cryo- and Freeze–Thaw	Strong and porous structures	Low with minimal chemical use	Moderate rates as low temperature is ideal for storage	Strong cell mimicry and drug delivery
Composite	Strong, porous, and tunable surfaces	Low–moderate with potential metal leakage	Slow rate of degradation	Strong cell mimicry and drug delivery

## Data Availability

No new data were created or analyzed in this study. Data sharing is not applicable to this article.
